# Supplementary material for: Exposure to Crude Oil-Related Volatile Organic Compounds Associated with Lung Function Decline in a Longitudinal Panel of Children
Source: Int J Environ Res Public Health. 2022 Nov 24;19(23):15599. doi: 10.3390/ijerph192315599 (PMC9737835; doi:10.3390/ijerph192315599)
Supplement: Supplementary file 1 [file ijerph-19-15599-s001.zip › ijerph-1990311-supplementary.pdf]

Supplementary Material

Table S1. Stratification by age in cross-sectional associations between exposure to VOCs and loss of lung function 1 year, 3 years, and 5 years after the very high environmental exposure to VOCs from the Hebei Spirit oil spill (n=224).

		**ppFEV$_1$**					
		**1 year later[a]**		**3 years later[b]**		**5 years later[c]**	
**Cumulative estimated concentration (mg/m^3·4 d)[d]**	**n (%)**	**β (SE)**	***p*-Value**	**β (SE)**	***p*-Value**	**β (SE)**	***p*-Value**
TVOCs[e]							
Age 4 to 5[f]	37 (16.5%)	-7.7 (4.4)	0.09	-10.1 (4.0)	0.02	-5.5 (4.1)	0.19
Age 6[f]	49 (21.9%)	-1.4 (2.5)	0.59	0.7 (2.9)	0.82	2.9 (2.4)	0.23
Age 7[f]	38 (17.0%)	-6.7 (2.8)	0.02	-12.7 (4.4)	0.007	-10.5 (3.9)	0.01
Age 8[f]	54 (24.1%)	-1.5 (2.3)	0.51	-8.0 (3.9)	0.05	-1.8 (3.5)	0.61
Age 9[f]	46 (20.5%)	-5.0 (2.4)	0.05	-0.2 (3.4)	0.95	-2.1 (3.5)	0.54
Benzene							
Age 4 to 5[f]	37 (16.5%)	-3.5 (6.2)	0.58	-15.0 (5.3)	0.008	-0.7 (5.7)	0.90
Age 6[f]	49 (21.9%)	-2.3 (3.6)	0.52	-1.9 (4.2)	0.66	4.6 (3.4)	0.18
Age 7[f]	38 (17.0%)	-3.6 (3.4)	0.29	-8.0 (5.3)	0.14	-11.4 (4.5)	0.02
Age 8[f]	54 (24.1%)	1.0 (2.7)	0.71	-1.0 (4.7)	0.83	3.3 (3.9)	0.41
Age 9[f]	46 (20.5%)	-4.4 (2.5)	0.08	-0.2 (3.3)	0.96	-5.0 (3.4)	0.15
Toluene							
Age 4 to 5[f]	37 (16.5%)	-7.2 (4.6)	0.12	-11.2 (4.1)	0.009	-4.8 (4.3)	0.26
Age 6[f]	49 (21.9%)	-1.6 (2.6)	0.53	0.2 (3.0)	0.95	3.1 (2.5)	0.22
Age 7[f]	38 (17.0%)	-5.8 (2.8)	0.05	-11.6 (4.4)	0.01	-10.6 (3.9)	0.01
Age 8[f]	54 (24.1%)	-1.1 (2.3)	0.63	-6.9 (4.0)	0.09	-0.9 (3.5)	0.80
Age 9[f]	46 (20.5%)	-4.9 (2.3)	0.04	-0.3 (3.2)	0.93	-2.6 (3.3)	0.42
Ethylbenzene							
Age 4 to 5[f]	37 (16.5%)	-7.7 (4.3)	0.08	-9.7 (3.9)	0.02	-5.5 (4.0)	0.19
Age 6[f]	49 (21.9%)	-1.3 (2.4)	0.60	0.8 (2.9)	0.79	2.8 (2.4)	0.24
Age 7[f]	38 (17.0%)	-6.8 (2.8)	0.02	-12.8 (4.4)	0.007	-10.3 (3.9)	0.01
Age 8[f]	54 (24.1%)	-1.6 (2.3)	0.49	-7.9 (3.9)	0.05	-1.9 (3.5)	0.59
Age 9[f]	46 (20.5%)	-4.9 (2.5)	0.05	-0.2 (3.4)	0.96	-2.1 (3.5)	0.55
Xylene							
Age 4 to 5[f]	37 (16.5%)	-7.6 (4.4)	0.09	-10.1 (4.0)	0.02	-5.3 (4.1)	0.20
Age 6[f]	49 (21.9%)	-1.4 (2.5)	0.58	0.6 (2.9)	0.84	2.9 (2.4)	0.23
Age 7[f]	38 (17.0%)	-6.5 (2.8)	0.02	-12.4 (4.4)	0.008	-10.4 (3.9)	0.01
Age 8[f]	54 (24.1%)	-1.5 (2.3)	0.52	-7.6 (3.9)	0.05	-1.6 (3.5)	0.65

Age 9[f]	46 (20.5%)	-4.9 (2.4)	0.05	-0.2 (3.3)	0.96	-2.3 (3.4)	0.51

ppFEV$_1$: percent predicted FEV$_1$.

Log scale of TVOCs, benzene, toluene, ethylbenzene, and xylene were used as the independent variable.

Estimates and *p*-Values were from generalized linear regression analysis adjusted for current smoking (no, yes) and second-hand smoke at home (no, yes).

[a]First survey, 2009; [b]Second survey, 2011; [c]Third survey, 2013.

[d]Cumulative estimated concentrations over the first four days after the oil spill were used.

[e]TVOCs: total volatile organic compounds including benzene, toluene, ethylbenzene, xylene, paraffin (C6–C12), cycloparaffin (C6–C12), and aromatic mono- and dicyclic components (C6–C11).

[f]Age at the time of the exposure to VOCs (age 4: n=4; age 5: n=33; age 6: n=49; age 7: n=38; age 8: n=54; age 9: n=46).

Table S2. Stratification by age in longitudinal associations between exposure to VOCs and loss of lung function up to 5 years after the very high environmental exposure to VOCs from the Hebei Spirit oil spill (n=224).

Cumulative estimated concentration ($mg/m^3 \cdot 4$ d)[a]	n (%)	ppFEV$_1$ β (SE)	ppFEV$_1$ *p*-Value
TVOCs[b]			
Age 4 to 5[c]	37 (16.5%)	-8.0 (2.9)	0.009
Age 6[c]	49 (21.9%)	1.1 (2.1)	0.59
Age 7[c]	38 (17.0%)	-8.8 (2.4)	0.001
Age 8[c]	54 (24.1%)	-1.7 (2.3)	0.47
Age 9[c]	46 (20.5%)	-4.5 (2.4)	0.07
Benzene			
Age 4 to 5[c]	37 (16.5%)	-8.4 (4.0)	0.05
Age 6[c]	49 (21.9%)	1.8 (3.0)	0.55
Age 7[c]	38 (17.0%)	-5.8 (3.0)	0.07
Age 8[c]	54 (24.1%)	1.2 (2.6)	0.66
Age 9[c]	46 (20.5%)	-4.5 (2.4)	0.06
Toluene			
Age 4 to 5[c]	37 (16.5%)	-8.3 (2.9)	0.008
Age 6[c]	49 (21.9%)	1.2 (2.2)	0.59
Age 7[c]	38 (17.0%)	-8.1 (2.5)	0.002
Age 8[c]	54 (24.1%)	-1.2 (2.3)	0.61
Age 9[c]	46 (20.5%)	-4.5 (2.3)	0.05
Ethylbenzene			
Age 4 to 5[c]	37 (16.5%)	-7.8 (2.8)	0.009
Age 6[c]	49 (21.9%)	1.1 (2.1)	0.59
Age 7[c]	38 (17.0%)	-8.8 (2.4)	0.0008
Age 8[c]	54 (24.1%)	-1.7 (2.3)	0.45
Age 9[c]	46 (20.5%)	-4.4 (2.4)	0.07
Xylene			
Age 4 to 5[c]	37 (16.5%)	-7.9 (2.8)	0.009
Age 6[c]	49 (21.9%)	1.1 (2.1)	0.59
Age 7[c]	38 (17.0%)	-8.6 (2.4)	0.001
Age 8[c]	54 (24.1%)	-1.6 (2.3)	0.49
Age 9[c]	46 (20.5%)	-4.4 (2.4)	0.07

ppFEV$_1$: percent predicted FEV$_1$.

Log scale of TVOCs, benzene, toluene, ethylbenzene, and xylene were used as the independent variable.

Linear mixed models after adjusting for smoking (no, yes), second-hand smoke at home (no, yes), and visit time (first survey, second survey, third survey) were used with an unstructured covariance matrix.

[a]Cumulative estimated concentrations over the first four days after the oil spill were used.

[b]TVOCs: total volatile organic compounds including benzene, toluene, ethylbenzene, xylene, paraffin (C6–C12), cycloparaffin (C6–C12), and aromatic mono- and dicyclic components (C6–C11).

[c]Age at the time of the exposure to VOCs (age 4: n=4, age 5: n=33, age 6: n=49, age 7: n=38, age 8: n=54, age 9: n=46; participated in all three surveys).
